# Defining Risk Drinking

**Published:** 2011

**Authors:** Deborah A. Dawson

**Keywords:** Alcohol consumption, alcohol use disorder, alcohol-related harm, alcohol and other drug use (AOD) use harm reduction, harm minimization, prevention of harm from AOD use, problematic AOD use, prevention of problematic AOD use, AOD induced risk, attributable risk, risk thresholds, morbidity, AOD risk mortality, AOD risk injury, standard drink, amount of AOD use, responsible AOD use

## Abstract

Many efforts to prevent alcohol-related harm are aimed at reducing risk drinking. This article outlines the many conceptual and methodological challenges to defining risk drinking. It summarizes recent evidence regarding associations of various aspects of alcohol consumption with chronic and acute alcohol-related harms, including mortality, morbidity, injury, and alcohol use disorders, and summarizes the study designs most appropriate to defining risk thresholds for these types of harm. In addition, it presents an international overview of low-risk drinking guidelines from more than 20 countries, illustrating the wide range of interpretations of the scientific evidence related to risk drinking. This article also explores the impact of drink size on defining risk drinking and describes variation in what is considered to be a standard drink across populations. Actual and standard drink sizes differ in the United States, and this discrepancy affects definitions of risk drinking and prevention efforts.

Preventing alcohol-related harm does not necessarily require that risk drinking be defined. At the population level, harm reduction can be achieved through numerous broad measures that determine the price or availability of beverage alcohol (Babor et al. 2003). Measures such as these affect drinkers at all consumption levels. Although there is inconsistent evidence as to whether their impact is greater among heavy or light-to-moderate drinkers (Farrell et al. 2003; Gmel et al. 2008; Heeb et al. 2003; Mäkelä et al. 2008; Manning et al. 1995; Wagenaar et al. 2009), such measures have proven to be effective in reducing problems associated with heavy or problem drinking (Wagenaar et al. 2009, 2010). In contrast to such global approaches, targeted approaches focus on preventing, identifying, and modifying risk drinking (i.e., drinking at levels or in patterns that increase the risk of alcohol-related harm). The development and dissemination of drinking guidelines that define the limits of low-risk alcohol consumption are one example of this type of prevention effort. Defining risk drinking may seem simple compared with preventing it, but in fact there are many conceptual and methodological challenges to arriving at a definition of risk drinking.

Perhaps the most essential challenge lies in determining the threshold that discriminates “low-risk” and “risk” drinking. Is risk drinking any consumption that corresponds to a significantly higher level of harm than that experienced by lifetime abstainers, or does the harm have to be of a specified magnitude? Given a linear relationship between consumption and harm, where is the appropriate cutoff point? Beyond this basic question, one must also ask what types of harms should be considered. Excessive use of alcohol is associated with a wide range of harmful outcomes, including alcohol use disorders; mortality and morbidity from chronic medical conditions, such as alcoholic liver disease, and acute causes, such as vehicular crashes and accidental and intentional injury; and a host of social and legal problems. Should risk-drinking definitions be keyed more closely to those types of harm most strongly attributable to alcohol use, or to the most severe harms (i.e., mortality or years of life lost) regardless of the strength of their association with drinking?

What aspects of alcohol consumption should be used to define risk drinking? Should these vary according to the type of harm (e.g., drinking volume in relation to chronic conditions, and drinking pattern in relation to acute alcohol-related harm)? Should risk drinking be defined in terms of consumption that reflects current alcohol-related problems, as is the case with screening for alcohol use disorders and emergency-department studies of drinking in relation to the risk of injury? Or should it be defined in terms of consumption that increases the risk of developing alcohol-related harm in the long term, as is the case with prospective studies of alcohol-related mortality and morbidity?

What types of studies are most appropriate for assessing associations between different aspects of alcohol consumption and alcohol-related harm? To what extent should we account for the quality of the consumption data upon which evidence of alcohol-related harm is based? Many of the large prospective studies used to assess mortality risk collect data on numerous putative risk factors, and they often contain too few questions on alcohol use to yield estimates of consumption that fully capture the contribution of heavy drinking days or multiple beverage types. If it is likely that associations of consumption with the risk of harm are based on underestimates of consumption, how should we account for that fact when using the data to inform definitions of risk drinking?

Finally, what is the appropriate cutoff between enough information and too much? Should definitions of risk drinking, or, conversely, low-risk drinking guidelines, be complex enough to include volume- and pattern-related risks and their variation across population subgroups or should they be simple enough so that drinkers can easily recall them and clinicians can easily identify risk drinkers based on a single metric? Many guidelines contain different limits for men and women; others stipulate lower limits for the youngest and oldest drinkers. In addition, some guidelines explicitly mention groups of individuals for whom any drinking is inadvisable (e.g., women who are pregnant or trying to become pregnant, people intending to drive or operate complex machinery, or individuals with medical conditions or taking medication). These exceptions to the general guidelines might also include individuals with former alcohol problems or those with a history of treatment for alcohol use disorders. Is it appropriate to use the same definition of risk drinking for all prevention efforts, or should the context determine the relative emphasis on different aspects of risk drinking? How should we account for variation across beverages and drinkers in drink size and alcohol content when defining risk drinking?

These questions provide some notion of the complex challenges posed in defining risk drinking and illustrate why there is no absolute consensus on the most appropriate definition. The following sections describe the evidence for associations of drinking volume and pattern with alcohol-related harm, issues surrounding standard drink size, and the conclusions drawn by selected countries in defining risk drinking in their national drinking guidelines.

## Association of Drinking Volume With Alcohol-Related Harm

Drinking volume, generally characterized in terms of average daily volume (ADV) of alcohol intake, has been widely studied in association with mortality and chronic disease morbidity in large prospective cohort studies. Because a full review of this extensive literature lies beyond the scope of this article, this section will summarize the findings of selected meta-analyses conducted in 2000 or more recently. The focus will be on studies with dose-response curves that can be used to inform risk-drinking definitions, rather than studies that summarize associations by means of alcohol-attributable fractions (i.e., the proportions of deaths from selected causes attributable to drinking) or years of life lost (e.g., Gutjarh et al. 2001; Rehm et al. 2003, 2006, 2007, 2009). Moreover, because the focus is on the definition of risk drinking, this article will not examine levels of drinking volume for which supposedly protective effects (i.e., risk levels lower than those of abstainers) have been observed.

Di Castelnuovo and colleagues (2006) conducted a meta-analysis of 34 prospective studies of all-cause mortality and established 56 independent risk curves reflecting various models and population subgroups. Visual inspection of the pooled adjusted risk curves for studies that presented both unadjusted and adjusted associations, including confidence intervals, suggests that the mortality risk began to significantly exceed the level of nondrinkers at an ADV of approximately 38 g of ethanol (or about 2.7 standard drinks). A similar threshold was observed for pooled studies that excluded occasional and former drinkers from the reference group, irrespective of the level of adjustment. Gender-specific curves, regardless of the level of control or reference group characteristics, showed that the risk of all-cause mortality started to increase at a lower ADV for women (approximately 35 g) than men (approximately 45 g). However, it has been argued that exclusion of studies biased by misclassification error with respect to the abstainer category might have yielded lower thresholds for harm (Fillmore et al. 2007). In a meta-analysis of all-cause mortality studies conducted before 2000, Gmel and colleagues (2003) reported a significant increase in risk relative to lifetime abstainers at ADVs of 30 g to 50 g for women (relative risk^1^ [RR]: 1.40) and at ADVs of 40 g to 70 g for men (RR: 1.04), based on studies where the mean age of the respondents was at least 45 years at baseline.

In terms of disease-specific morbidity and mortality, much of the literature concerning coronary heart disease (CHD), stroke, and type 2 diabetes has centered on the debate concerning possible protective effects of moderate volumes of intake, but some meta-analyses have reported increased risks of these diseases at heavy volumes of consumption. A meta-analysis of 28 CHD studies by Corrao and colleagues (2004) indicated an increased risk compared with nondrinkers at an ADV of 89 g. Corrao and colleagues observed increased risks of hemorrhagic stroke at ADVs of 50 g and higher in the meta-analyses of six studies and of ischemic stroke at an ADV of 100 g in the meta-analyses of three studies. These findings for stroke risk are consistent with those of Reynolds and colleagues (2003), who conducted a meta-analysis of 35 cohort and case-control studies of stroke risk. They reported a significantly increased risk of all types of stroke at ADVs of 60 g and higher, with an RR that was twice as great for women as men (RR: 4.29 vs. RR: 1.76) at those volumes of intake. With respect to type 2 diabetes, results of meta-analyses have been inconsistent. At low thresholds for heavy drinking, neither Koppes and colleagues (2005) nor Carlsson and colleagues (2005) found any increased risk. However, a more recent meta-analysis by Baulina and colleagues (2009) of 20 cohort studies found that the risk of type 2 diabetes increased for men at an ADV of approximately 60 g and for women at an ADV of approximately 50 g.

Corrao and colleagues (2004) reported a linear dose-response function for essential hypertension on the basis of a meta-analysis of two studies, with significantly increased risks corresponding to ADVs as low as 25 g (RR: 1.43, increasing to 2.04 at an ADV of 50 g and to 4.15 at an ADV of 100 g). In a larger meta-analysis of 12 cohort studies, Taylor and colleagues (2009) reported significant RRs of 1.57 for men and 1.81 for women at an ADV of 50 g and of 2.47 for men and 2.82 for women at an ADV of 100 g. Neither of these meta-analyses reported specific cut points at which the risk of hypertension was increased. Other nonneoplastic conditions (i.e., non-cancerous conditions) for which linear dose functions were observed, at least up to an AVD of 100 g, included chronic pancreatitis and liver cirrhosis. For both of these conditions, the risk was significantly increased at an ADV of 25 g (RR: 1.34 and 2.90, respectively; Corrao et al. 2004). In one prospective study that presented gender-specific risk curves, the risk of all types of liver disease increased at a lower volume of alcohol intake for women (7 to 13 drinks per week) than men (14 to 27 drinks per week).

In a meta-analysis of six studies of pancreatitis, Irving and colleagues (2009) reported a monotonic and approximately exponential dose-response relationship between the ADV and the risk of pancreatitis. On the basis of a continuous risk curve, the risk of pancreatitis was significantly increased at an ADV of 36 g (RR: 1.2), but categorical models found that the association was not significantly increased until the ADV reached levels greater than 48 g (RR: 2.5). Chong and colleagues (2008), who conducted a meta-analysis of seven cohort studies examining alcohol consumption and age-related macular degeneration, found an increased risk of early-stage macular degeneration at an ADV of greater than 30 g (odds ratio [OR]: 1.47); however, the association with ADV was not significant for late-stage macular degeneration.

Finally, in terms of neoplastic conditions, Corrao and colleagues (2004) reported significantly increased risks of oral, pharyngeal, esophageal, laryngeal, colon, rectal, liver, and breast cancers at ADVs of 25 g and greater. Other meta-analyses have reported RRs for laryngeal cancer relative to nondrinkers of 1.94 at an ADV of 50 g and of 3.95 at an ADV of 100 g (Altieri et al. 2005) and RRs for colorectal cancer of 1.41 at ADVs of 45 g and greater, with an RR of 1.16 that fell just short of significance for ADVs of 30 g to 44 g (Cho et al. 2004). Increased risks of breast cancer also have been reported at an ADV of 12 g (RR: 1.06; Ellison et al. 2001) and 35 g to 44 g (RR: 1.32; Hamajima et al. 2002).

## Association of Drinking Pattern With Alcohol-Related Harm

For some types of alcohol-related harm, notably those that reflect acute consequences of heavy-drinking occasions, average volume of intake is a less relevant risk factor than measures of alcohol use directly associated with the event (i.e., drinking in the event) or of heavy episodic drinking patterns. Drinking in the event is typically measured by means of a positive blood alcohol content (BAC) result or self-report of drinking in the 6 hours preceding an injury or medical problem. Heavy episodic drinking (HED), sometimes called risky single-occasion drinking (RSOD; Gmel et al. 2011) or binge drinking, traditionally has been defined—at least in the United States—as consuming five or more drinks in a single day or a single drinking occasion. However, a definition based on five or more drinks for men and four or more drinks for women (Wechsler and Nelson 2001; Wechsler et al. 1995) has come into increasing use in recent decades. Although the scientific basis for defining HED as five or more drinks is somewhat obscure, the definition of five or more drinks (for men) or four or more drinks (for women) is supported by its close correlation with the amount of ethanol required to achieve a BAC of 0.75 g to 0.80 g per kg of body weight (Dawson et al. 1996). Such concentrations have been shown to be associated with psychomotor and cognitive impairment in experimental studies (Hindenmarch et al. 1991; Lane et al. 2004). The associations of harm with drinking in the event and HED measures have been assessed using a wide variety of study designs, including case-control, case crossover, and experimental blood alcohol level dose-response studies, in addition to cross-sectional and prospective analyses of general population data.

Emergency-department studies have been a major source of data on the association between drinking and the likelihood of injury severe enough to warrant treatment at an emergency department (see the review in Cherpitel 2007). Case-control studies, which examine the odds of presenting at an emergency department with an injury as opposed to a non–injury-related medical problem, have shown that the odds of injury are increased at even low volumes of consumption (e.g., one drink a week) and with any frequency of drinking five or more drinks on any single occasion. However, the risk curves from the Emergency Room Collaborative Alcohol Analysis Project (ERCAAP) showed a tendency to level off at volumes of ethanol intake greater than two drinks per day, or drinking five or more drinks on any single occasion more often than monthly. Although the shapes of the risk curves were similar for men and women, injury risks were lower at comparable drinking (Cherpitel et al. 2006). In contrast, an Australian case-control study of individuals hospitalized for injury paired with community control subjects found significantly higher risks of injury among women with an in-the-event intake of more than 60 g of ethanol (Stockwell et al. 2002). In general, the risk of injury is more strongly associated with drinking in the event than with regular drinking patterns. In fact, a meta-analysis of emergency-department studies demonstrated that pooled attributable risk sizes were 43 percent for drinking in the event compared with just 27 percent for usual drinking pattern (Cherpitel et al. 2005). Pooled data from the ERCAAP showed that individuals who tested positive for drinking in the event were more than 50 percent more likely to present for an injury as opposed to a medical problem (Cherpitel et al. 2003).

Individual case crossover studies, in which an individual’s self-reported regular drinking pattern during some specified period is used as the “control” for his or her self-reported drinking in the event (Maclure 1991), have shown three- to fourfold increases in the risk of injury in association with drinking in the event (Borges et al. 2004; Vinson et al. 2003*a*). One study showed the excess risk increasing directly with the number of drinks consumed from an OR of 1.8 for one to two drinks to an OR of 17.0 for seven or more drinks (Vinson et al. 2003*b*). Pooled data from 28 emergency-department studies in 16 countries showed that the random pooled effect of drinking in the event compared with usual drinking was an increase of 5.69 in the likelihood of injury (Borges et al. 2006). Associations with drinking in the event are even stronger for violence-related injuries than for all injuries, with a case crossover study showing a 34-fold increase in the risk for a violence-related injury associated with drinking in the event relative to drinking the previous day and a 10-fold increase relative to drinking in the previous month (Vinson et al. 2003*a*). The ERCAAP data also indicated that the odds of a violence-related injury as opposed to an unintentional one were increased by a factor of 5.5 at BACs of 0.15 to 0.199 (Macdonald et al. 2005). Evidence of gender differences was mixed. Associations of drinking in the event with violent versus unintentional injury were greater for men than women in Argentina, Belarus, and Spain but greater for women than for men in China. There were no gender differences with respect to drinking in the event in the United States, but the association between frequent HED and violent injury was greater among American women than men (OR: 4.52 vs. 1.63) (Wells et al. 2007).

Because deaths from external causes generally reflect drinking in the event of a fatal injury, analyses of the role of alcohol in such deaths have focused more strongly on drinking pattern than average volume of ethanol intake. In a prospective study of Russian men aged 25 to 64 years, with an average follow-up of 9.5 years, usual consumption of at least 160 g of alcohol per occasion increased the risk of death from external causes by a factor of 2.08 compared with a usual consumption of less than 80 g among individuals who drank at least once a month. A similar prospective study of Finnish men found that those who usually drank six or more bottles of beer per drinking occasion had a far higher risk of death from external causes than those who usually consumed less than three bottles (RR: 7.10), even after adjusting for total consumption (Kauhanen et al. 1997). No significant increase in risk was observed at lower usual levels of intake (Malyutina et al. 2002). In a study of fatal injury that entailed matching death records with data from a series of Finnish alcohol surveys, consuming five or more drinks 25 to 52 times per year and more than 52 times per year were associated with fatal injury RRs of 2.63 and 5.78, respectively, relative to never consuming five or more drinks, even after adjusting for frequencies of drinking fewer than five drinks (Paljärvi et al. 2005). Dawson (2001) also found an increased risk of mortality from external causes in association with usual consumption of five or more drinks among U.S. adults, but only among those who drank this amount less than once a month. In a study of single-vehicle motor vehicle crashes, Heng and colleagues (2006) reported that the risk of fatality was significantly increased even at BACs associated with fairly low levels of in-the-event consumption (e.g., at BACs as low as 0.010 to 0.019 for drinkers ages 16 to 20).

Although most studies of chronic disease and all-cause mortality have focused on the association with volume of ethanol intake, as described previously, a limited number of studies have examined associations with drinking pattern measures, primarily HED. Tolsrup and colleagues (2004) found that the all-cause mortality risk associated with drinking 21 or more drinks a week was greater among people with infrequent as opposed to frequent intake (the former implying more drinks per drinking occasion) in a prospective Danish cohort study. On the basis of a Finnish cohort study of men ages 25 to 64, Laatikainen and colleagues (2003) found that the prospective risk of all-cause mortality was 57 percent higher among men who had consumed six or more drinks at a time than among those who had not, even after controlling for volume of consumption. Another Finnish cohort study reported that men who usually drank six or more beers per occasion had higher risks of all-cause mortality and fatal myocardial infarction than those who usually consumed fewer than three beers (RR: 3.01 and 6.50, respectively), independent of their total volume of consumption (Kauhanen et al. 1997). Mäkelä and colleagues (2005), who linked Finnish alcohol survey participants with death records, found an increased risk of all-cause mortality among men in association with a high volume of ethanol intake consumed on heavy-drinking occasions, but not in association with a high volume consumed on lighter-drinking occasions. This relationship did not extend to women. In a 3.8-year follow-up of patients hospitalized for myocardial infarction, those who had consumed three or more drinks within a 1- to 2-hour period at least once in the past year were twice as likely to have died as those who had not (OR: 2.1 after adjusting for usual alcohol intake; Mukamal et al. 2005). Rivara and colleagues (2004), who applied etiologic fractions (the proportion of the cases caused by exposure) for alcohol-attributable mortality to data from a series of cross-sectional surveys of the U.S. general population, reported that more than one-half of the deaths attributed to harmful drinking in the United States were a result of HED rather than medium to high volumes of intake.

In a meta-analysis of six studies that included drinking pattern and volume measures, Bagnardi and colleagues (2008) found an increased risk of cardiovascular disease among drinkers compared with nondrinkers at a weekly average of 131 g among individuals who drank twice a week or less often (implying an intake of at least 65.5 g on drinking days). This same study did not find an increased CHD risk at even the highest weekly volumes among those who drank more regularly. In a cross-sectional analysis of drinking pattern and the prevalence of coronary calcification at the 15-year follow-up of a cohort study of young adults ages 18 to 30 at baseline, having consumed five or more drinks at least once in the past month was associated with a significantly increased risk of calcification (OR: 2.1) independent of volume of intake. In an 8-year follow-up of a Canadian population sample, the hazard rate of CHD mortality and morbidity was significantly increased among men (hazard rate ratio^2^ [HRR]: 2.26) and women (HRR: 1.10) who had consumed eight or more drinks on at least one occasion in the past year, independent of mean volume of intake. For men only, consumption of eight or more drinks in the past year also was associated with a modest increase in morbidity and mortality from hypertension (HRR: 1.57; Murray et al. 2002). A Finnish cohort study of twins followed for 25 years found that monthly or more frequent consumption of five or more drinks in midlife was associated with a more than threefold increase in the risk of developing dementia (Jarvenpaa et al. 2005).

Among the other types of alcohol-related harm that have been associated with HED or high BAC concentrations in experimental, cross-sectional, and prospective surveys are violence (Brewer and Swahn 2005), including intimate-partner violence (see reviews in Foran and O’Leary 2008; Marshal 2003) and other forms of victimization (e.g., Connor et al. 2009; Stickley and Pridemore 2009; Testa and Livingston 2009; Wells and Thompson 2008); social and legal problems (e.g., Dawson et al. 2008: Rehm and Gmel 1999; Viner and Taylor 2007; Wechsler and Nelson 2001); physical and mental quality of life (Green et al. 2004; Okoro et al. 2004); various aspects of cognitive functioning, including impaired judgment and risk taking (Breitmeier et al. 2007: Cairney et al. 2007; Goudriaan et al. 2007; Lane et al. 2004; Neal and Fromme 2007); and fetal alcohol spectrum disorders (FASD) resulting from maternal drinking during pregnancy (Bailey and Sokol 2008; Testa et al. 2003). Consistent with the previously cited studies, these studies generally reported linear risk curves, sometimes with a threshold effect and often with significantly increased risks at relatively low frequencies of HED or usual/in-the-event quantities of drinks consumed.

## Associations of Drinking Volume and Pattern With Alcohol Use Disorders

Associations of drinking volume and pattern with alcohol use disorders (AUDs) have been established in both cross-sectional and prospective designs. Studies of general-population samples have shown that both the average volume of consumption and the absolute or relative frequency of heavy drinking are independently associated with the risk of alcohol abuse and dependence (e.g., see Caetano et al. 1997; Dawson and Archer 1993; Dawson et al. 1995; Midanik et al. 1996; Rehm et al. 2005). In a study of U.S. past-year drinkers that examined daily drinking limits of no more than four drinks for men and three drinks for women and weekly drinking limits of no more than 14 drinks for men and 7 drinks for women, where drinks were defined as the equivalent of 0.6 oz of ethanol, the prevalence of alcohol dependence showed a linear increase with the frequency of exceeding the daily limits. At some, but not all, frequencies, the prevalence of dependence also was significantly higher among individuals who exceeded the weekly limits than those who did not (Dawson et al. 2005*a*). Over the course of a 3-year follow-up interval, a prospective study of U.S. adults revealed that baseline frequency of drinking five or more standard 0.6-oz drinks (for men) or four or more (for women) had a positive linear association with the first incidence of alcohol abuse and dependence that was significant even after controlling for ADV of ethanol intake (Dawson et al. 2008). Individuals who consumed five or more drinks (men) or four or more drinks (women) on a daily or near-daily basis at the baseline interview had an almost fourfold increase in the odds of incident alcohol abuse and more than a sevenfold increase in the odds of incident dependence.

The criteria in the *Diagnostic and Statistical Manual of Mental Disorders, Fourth Edition* (DSM–IV; American Psychiatric Association 1994) for alcohol use disorders do not require any minimum level of consumption for a positive classification of abuse or dependence, although a consumption criterion has been proposed as one possible means to tap the less severe range of the latent trait of AUDs (Saha et al. 2007). Rather, alcohol consumption is viewed as a correlate of AUD (both a precursor and an outcome, given the complex reciprocal relationship between the two), and consumption questions have come into increasing use in brief-screening instruments designed to identify individuals with AUDs in primary and emergency care settings. Several brief screeners containing both alcohol consumption and alcohol problem items have shown high levels of sensitivity (the proportion of individuals with the AUD outcome in question who screen positive) and specificity (the proportion of individuals without the AUD outcome in question who screen negative) for AUD and/or hazardous drinking (Berner et al. 2007; Kelley et al. 2009). These include the Alcohol Use Disorders Identification Test (AUDIT; Saunders et al. 1993) and the Rapid Alcohol Problems Screen-Quantity Frequency (RAPS4-QF; Cherpitel 2002). The AUDIT-C, containing only the three AUDIT consumption questions on overall frequency of drinking, usual quantity of drinks consumed on drinking days, and frequency of heavy drinking, has proven nearly as effective as the full 10-question AUDIT in screening for AUDs and risk drinking in the general population and subpopulations such as veterans and patient samples (Aertgeerts et al. 2001; Bradley et al. 2003; Bush et al. 1998; Dawson et al. 2005*b*; Gordon et al. 2001; Gual et al. 2002; Rumpf et al. 2002). More recently, studies of a single-item screening instrument based solely on the frequency of heavy drinking also have reported high levels of sensitivity and specificity in screening for AUDs and risk drinking in trauma center and emergency-department samples (Canagasaby and Vinson 2005; Dawson et al. 2010; Seale et al. 2006; Smith et al. 2009; Stewart et al. 2008; Taj et al. 1998; Williams and Vinson 2001). In a sample of U.S. adults, drinking five or more drinks (men) or four or more drinks (women) at least once in the preceding year resulted in a sensitivity and specificity of 86.7 percent and 82.1 percent, respectively, in predicting DSM–IV alcohol abuse and/or dependence (Dawson et al. 2010). Many of the studies of the AUDIT, AUDIT-C, and other brief screening instruments have noted differential performance across subgroups of the general U.S. population, often supporting lower screening score thresholds for detecting problem drinking among women and the elderly (Berner et al. 2007; Dawson et al. 2005, in press; Kelly et al. 2009). A recent test of a single-item screener based on maximum drinks consumed likewise found variation across subgroups. By gender, the cut point that maximized sensitivity and specificity for any AUD or any AUD/hazardous drinking was five or more drinks for men and four or more drinks for women (Dawson et al., in press), thus providing support for the gender-specific five or more/four or more drinks definition of risk drinking that has come into common use in U.S. surveys of drinking practices and problems (Wechsler and Nelson 2001; Wechsler et al. 1995).

## International Low-Risk Drinking Guidelines

Perhaps the best illustration of the complexity of defining risk drinking can be obtained by comparing international drinking guidelines. A number of countries have systematically formulated low-risk drinking guidelines with the input of expert committees of researchers who have conducted extensive reviews of the scientific literature. By providing the upper limits of low-risk drinking, these guidelines implicitly reveal various countries’ definitions of risk drinking (i.e., consumption beyond the low-risk limits). Two reports describing recent changes to the Australian and Canadian drinking guidelines provide the rationales used for setting the guidelines in those countries. They illustrate the broad range of evidence typically considered in establishing these guidelines and the diverse approaches (e.g., relative versus absolute risk) that may be applied to the interpretation of this evidence (National Health and Medical Research Council, http://www.nhmrc.gov.au/_files_nhmrc/file/publications/synopses/ds10-alcohol.pdf; Stockwell et al., in press).

Low-risk drinking guidelines vary substantially across countries, as is evident in an online listing of these guidelines that is continually updated (see http://www.icap.org/Publications/ICAPReports/tabid/75/Default.aspx). The various guidelines differ among countries not only in terms of the maximum permissible numbers of drinks but also in terms of what types of limits are included (daily, weekly, or both) and whether there are different guidelines for men and women (see table). Although most countries’ drinking guidelines are expressed solely in terms of daily limits, Denmark, Finland, Ireland, and South Africa have weekly drinking limits only. Canada, New Zealand, Poland, the United Kingdom, and the United States include both daily and weekly limits. Australia and Slovenia provide limits for consumption on “any day” (which when multiplied times seven roughly correspond to weekly limits), with higher limits for any single drinking occasion. The Australian guidelines explicitly state that the former are targeting the risk of chronic conditions and the latter the acute consequences of heavy drinking. The United States, Canada, Italy, and South Africa all have definitions of moderate drinking that are more restrictive than their thresholds for low-risk drinking, and Spain’s guidelines include regional variations (i.e., considerably higher limits in the Basque country and Catalonia than in the overall national guidelines). In general, low-risk drinking guidelines stipulate the upper limit of low-risk consumption, but a few countries (i.e., Japan, Portugal, Romania, Spain [Catalonia], the United Kingdom, and the United States [Dietary Guidelines moderate drinking definition for men]) provide a range of acceptable values.

Many of the differences across countries in the specific numbers of drinks comprising daily or weekly limits reflect variation in the standard drink size used to express the daily and/or weekly limits. The standard drink size assumed by the U.S. drinking guidelines (0.6 oz or approximately 14 g of ethanol) is almost twice as large as the standard drink size of 8 g used by the United Kingdom. A standard drink size of 10 g is used in Australia and most of Europe other than the United Kingdom; the Canadian standard drink size is 13.45 g. Japan lies at the upper extreme, with a standard drink size of 19.75 g. For a full description of international variation in standard drink size, see http://www.icap.org/table/InternationalDrinking Guidelines and Devos-Comby and Lange (2008). However, even when expressed in the common metric of grams per day or week, the upper limits of low-risk drinking vary considerably across countries. Daily limits for men vary from a low of 20 g in Poland and Sweden to a high of 70 g in the Basque country of Spain and those for women vary from 10 g in Poland to 70 g in the Basque country. Weekly limits for men lie in the range of 100 g in Poland to 252 g in Denmark and South Africa, whereas those for women lie in the range of 50 g in Poland to 168 g in Denmark and South Africa.

Australia, Canada, France, Italy, Romania, Singapore, Spain (all regions), Sweden, and Switzerland have identical low-risk drinking guidelines for men and women, although Italy also has a set of Nutritional Guidelines with complex moderate drinking recommendations based on total body weight that generally would yield higher limits for men than women. The Canadian guidelines from the Center for Addiction and Mental Health have identical daily limits for men and women but lower weekly limits for women. Japan has guidelines for men only. The remaining countries for which guidelines are available—Austria, Canada (National Alcohol Strategy Advisory Committee), the Czech Republic, Denmark, Finland, Germany, Ireland, the Netherlands, Poland, Portugal, Slovenia, South Africa, the United Kingdom, and the United States) have different drinking limits for men and women in all components of their low-risk drinking guidelines. Typically, the gender differences in the daily limits are proportionately smaller than those in the weekly limits.

There have been surprisingly few attempts to validate individual countries’ drinking guidelines against specific outcomes. In a cross-sectional analysis of the U.S. drinking guidelines among past-year drinkers, Dawson (2000*a*) found that exceeding the weekly limits or having ever exceeded the daily limits in the past year yielded high sensitivity for alcohol dependence, impaired driving, liver disease, peptic ulcer, and hypertension (64.3 percent to 98.5 percent), but that specificity was extremely low (28.7 percent to 32.5 percent) for these outcomes. Specificity was improved (72.0 percent to 76.1 percent) at a higher frequency of exceeding the daily limits (at least once a week), but sensitivity was accordingly reduced (30.8 percent to 70.6 percent). In multivariate models adjusted for sociodemographic characteristics, the odds of all of the outcomes except for peptic ulcer were significantly increased among drinkers who exceeded the weekly or daily limits, regardless of the frequency of the latter. It is interesting to note that the odds ratios virtually were identical when considering only whether the drinkers exceeded the daily limits. That is, little additional information on risk was obtained by considering the weekly as well as daily limits. A more recent evaluation of the U.S. guidelines considered past-year alcohol consumption relative to multiple concurrent and prospective harms and found that the thresholds that optimized prediction of concurrent harm (i.e., the upper limits of what might be considered low-risk drinking) consisted of 4 drinks a day for men and 3 drinks a day for women (4/3), alone or in combination with weekly limits of 21 drinks for men and women. Prospective harms were best predicted by weekly limits of 14/7 (men and women, respectively), 14/14 and 10/10 drinks, all combined with daily limits of 4 drinks for both men and women (Dawson et al., in press). Using a prospective framework, Batty and colleagues (2009) recently examined the effect of exceeding the daily and weekly U.K. drinking limits on the occurrence of various harms over the course of a 3.6-year follow-up interval. They reported that exceeding the daily limits was associated with an increased risk of hypertension, whereas exceeding the weekly limits was associated with an increased risk of financial problems. Of interest is a near-significant association with accidents occurred with respect to exceeding the weekly rather than daily limits, suggesting that the in-the-event levels of consumption typically associated with injuries did not significantly increase the risk of accidents unless they were consumed often enough to yield a volume of intake that exceeded the weekly drinking limits.

## The Effect of Drink Size on Definitions of Risk Drinking

Despite the abundant evidence of chronic and acute alcohol-related harm at various levels of average daily or per-occasion ethanol intake, converting risk thresholds into a comprehensible definition of risk drinking must ultimately confront the issue of drink size. The amount of ethanol contained in an alcoholic drink varies considerably depending on the type of alcohol (e.g., beer, wine, or spirits) and on the size of the drink. Within the major categories of alcoholic beverages, there are significant variations according to beverage subtype. Malt liquor, with a typical ethanol content of at least 6.0 percent alcohol by volume (ABV), is far stronger than light beer, with a typical ABV of about 4.2 percent. Likewise, fortified wines have an ABV that is about 50 percent greater than that of regular table wine or champagne, approximately 18 percent versus 12 percent (Kerr et al. 2006*a*). Moreover, spirits such as whiskey, vodka, and gin have an ABV that is greater than that of cordials and liqueurs and far greater than that of prepackaged cocktails (Kerr et al. 2006*b*). To account for differences such as these, drinking guidelines often provide examples of what constitutes a standard drink. The National Institute on Alcohol Abuse and Alcoholism (NIAAA) low-risk drinking guidelines for the United States, for example, define a standard drink containing 0.6 oz or 14 g of ethanol as the equivalent of 12 oz of beer or wine cooler; 8 oz to9 oz of malt liquor; 5 oz of table wine; 3 oz to 4 oz of fortified wine; 2 oz to 3 oz of cordial, liqueur, or aperitif; and 1.5 oz of brandy, whiskey, vodka, etc. (NIAAA 2005; see also http://rethinkingdrinking.niaaa.nih.gov/). They also provide information on the number of standard drinks in commonly sold container sizes such as a 40-oz can of malt liquor, a 750-mL bottle of table wine, and a fifth of spirits.

Does this ensure that drinkers understand the concept of a standard drink and know how many standard drinks they typically consume? Unfortunately, all evidence suggests that this is not the case. In an exhaustive review of 32 studies related to drink size published through 2007, Devos-Comby and Lange (2008) found that drinkers often were unaware of how standard drinks were defined in their countries and that actual drink sizes (or attempts to pour a standard drink) often exceeded standard drink sizes. The magnitude of the discrepancy varied substantially across studies and was associated with study design, drinker characteristics, type of beverage, and vessel size. More recent U.S. research confirmed that vessel size was more important than shape in determining the size of drink pours (Kerr et al. 2009*a*) and that larger-than-standard drinks were common even in bar and restaurant drinks (Kerr et al. 2008). Although larger-than-standard drink sizes are the major concern in the prevention of risk drinking, it should be noted that a significant proportion of drinkers consume smaller-than-standard drinks. In fact, Kerr and colleagues (2005, 2009*b*) have shown that there is a great deal of dispersion in the distribution of actual drink sizes and that the degree of dispersion varies by beverage (smallest for beer and larger for wine and spirits) and by demographic characteristics (smaller for men and Whites and larger for women and minorities). Thus, it must be understood that many drinkers will interpret drinking guidelines in terms of numbers of drinks that correspond to levels of intake that are smaller or larger than those intended by the standard drink definitions included in the guidelines. In light of this, it might be argued that standard drink sizes for any given country should reflect the most common container or serve sizes in that country, even if this leads to lack of comparability across countries. That is, the standard drink definitions that maximize prevention efforts may not be those best suited for comparative research purposes. Research addressing how guidelines are understood by drinkers who typically pour non-standard drinks might help to improve the delivery of drinking guidelines to these individuals.

## Conclusions

Definitions of risk drinking, as implied by the low-risk drinking guidelines of the United States and other countries, are generally in line with levels of risk observed in the scientific literature. Although estimated associations of alcohol consumption with all-cause mortality and chronic disease vary as a function of level of adjustment and reference group, the ADV at which an increased risk of mortality is apparent generally lies in the range of 35 g to 45 g, or 245 g to 315 g per week, and the risk of many chronic medical conditions is significantly increased (albeit quite modestly in many cases) at ADVs as low as 25 g, or 175 g per week. The weekly drinking limits for the majority of countries lie within this range. Evidence for gender differences in the association of drinking volume and chronic harm is both sparse and inconsistent but suggests that risk thresholds may be somewhat lower for women than men, at least for some conditions. The quite substantial differences in men’s and women’s weekly drinking guidelines in a number of countries, including the United States, with limits of 196 g and 98 g, respectively, are not fully supported by the existing data. They are more consistent with an influential early analysis of alcohol and all-cause mortality conducted by English and colleagues (1995), which reported modest but significant increases in all-cause mortality at an ADV of 20 g for women compared with 40 g for men. It is important to bear in mind that many of the mortality and chronic- disease studies summarized previously were large prospective studies that collected information on numerous risk factors for disease and mortality. Estimated volume of ethanol intake may be based on minimal data and sometimes represents nothing more than the product of a single question on drinking frequency times a single question on usual or average number of drinks consumed on days when drunk. As a result, any given ADV is probably an underestimate, and the harm associated with specific ADV levels is therefore likely associated with what is actually a larger ADV.

Acute alcohol-related harm generally shows a linear increase with drinking in the event and frequency of HED, sometimes with a threshold effect (i.e., an attenuation or evening off of the slope of the risk curve after a certain number of drinks or HED frequency). Linear risk curves provide no obvious basis for determining the cutoff corresponding to risk drinking, which may help to explain the wide range of daily drinking limits in international low-risk drinking guidelines. Moreover, the data as related to gender differences in the association of acute alcohol-related harm and drinking are highly inconsistent across studies. In part, these inconsistencies may reflect gender differences in the underlying probability of the type of acute harm being studied, (e.g., the greater tendency of men to engage in violent behavior irrespective of drinking level). This raises questions as to whether risk-drinking definitions for men and women should reflect gender differences in the underlying harm probabilities themselves or rather in the extent to which these probabilities are modified by drinking (Dawson 2009). One solution to this issue is to key risk-drinking definitions (and low-risk drinking limits) to ethanol intake levels corresponding to psychomotor impairment, an approach that helped to inform the NIAAA low-risk daily drinking limits (see table). It is worth noting that the U.S. drinking limits, especially those for men, are among the highest internationally. In part, this may reflect the fact that the current standard drink size of 0.6 oz or 14 g, slightly larger than the standard drink size of approximately 12 g that was assumed at the time those guidelines were first drafted. In addition, the risk-drinking definition of five or more drinks (men) or four or more (women) underlying the NIAAA daily drinking limits has proven optimal in its ability to identify individuals with AUDs, an outcome of obvious importance to NIAAA (National Institute on Alcohol Abuse and Alcoholism 1998) and a type of alcohol-related harm that may have been given less weight in the construction of other countries’ drinking guidelines. One final note of caution is with respect to applying scientific evidence to daily drinking limits. The near-universal use of five or more drinks (men) or four or more drinks (women) as a measure of HED (or their equivalent in countries with smaller standard drink sizes) means that few studies have been able to evaluate whether some other measure of HED would be more strongly associated with harm. Likewise, when measures are inherently gender based, such as the definition of HED using five or more drinks (men) or four or more drinks (women), this precludes testing for the significance of gender differences in the relationship between HED and alcohol-related harm.

Questions about how best to convey the definition of risk drinking to the public remain even after evidence-based risk-drinking limits have been established. As is evident from a review of international drinking guidelines, the United States is among a minority of countries that define risk drinking both in terms of daily and weekly consumption. It is arguable that daily limits alone would be sufficient. The logic for such an argument is that most U.S. drinkers are not daily drinkers but rather consume alcohol primarily on weekends and special occasions (NIAAA 1998). Given such a pattern of intake, individuals who adhered to low-risk daily drinking limits would not exceed weekly limits simply because they would have too few drinking occasions to do so. However, drinking patterns vary over the life course, with most data indicating increasing frequency of drinking in lower quantities in relation to aging (Dawson 2000*b*). This being the case, weekly limits, or a definition of risk drinking that includes weekly consumption, may be useful for the growing number of drinkers aged 55 and older among the aging Baby Boomers in the United States.

Regarding the perplexing and challenging issue of drinkers’ inability to accurately gauge their consumption in standard drinks, the most obvious solution lies in the approach that has been adopted by a number of Western countries, in which alcoholic beverage containers explicitly state how many standard drinks (“units”) they contain. Even in the absence of such labeling, it has been argued that if risks attributed to drinking five or more drinks are based on scientific evidence relying on actual as opposed to standard drink sizes, coupled with other sources of consumption underreporting, then drinking less than five drinks, irrespective of how closely they correspond to standard drink size, will reduce harm in the aggregate. That is, if one assumes that relative risks associated with various consumption levels are overstated because of underreporting of consumption, then adherence to low-risk drinking limits should prove effective even for individuals whose actual drink sizes are larger than standard. Hence, publicizing low-risk drinking limits should play an important role in any activities aimed at preventing alcohol-related harm.

## Figures and Tables

**Table:** International Low-Risk Drinking Guidelines, Expressed in Grams

	**Daily drinking limits**		**Weekly drinking limits**	
	**Men**	**Women**	**Men**	**Women**
Australia	40	40	140^a^	140^b^
Austria	24	16		
Canada (Centre for Addiction & Mental Health)	27.2	27.2	190	121.5
Canada (National Alcohol Strategy Advisory Committee)	53.8^b^	40.4^b^	201.8^b^	134.5^b^
Czech Republic	24	16		
Denmark			252	168
Finland			165	110
France	30	30		
Germany	24	12		
Ireland			210	140
Italy	40	40		
Japan	19.8–39.5			
Netherlands	39.6	19.8		
New Zealand	30	20	210	140
Poland	20	10	100	50
Portugal	28–42^c^	14–28^c^		
Romania	32.5d/20.7^e^	32.5d/20.7^d^		
Singapore	30	30		
Slovenia	50	30	140^a^	70^a^
South Africa			252	168
Spain (general)	30	30		
Spain (Basque country)	70	70		
Spain (Catalonia)	32–50	32–50		
Sweden	20	20		
Switzerland	24	24		
United Kingdom	24–32	16–24		
United Kingdom (Scotland)	24–32	16–24		
United States (National Institute on Alcohol Abuse and Alcoholism)	56	42	196	98
United States (Dept. of Agriculture/Department of Health and Human Services)	14–28	14	196	98

a Based on multiplying upper limits to be consumed “on any day” times 7 (daily limits refer to a single drinking occasion)

b To be confirmed

c Limits refer to wine only

d Beer

e Wine

SOURCE: International Center for Alcohol Policy, http://www.icap.org/Publications/ICAPReports/tabid/75/Default.aspx

## Footnotes

1 Relative risk is a ratio of the probability of the event occurring in the exposed group versus a nonexposed group. For example, a relative risk of 10 means that the exposed group is 10 times more likely than the nonexposed group to experience the event.

2 Hazard rate ratio measures the likelihood of an outcome occurring at a specific time point among those still at risk. The ratio is assumed to be constant/proportional over time points.
